# Nerve Imaging, Electrodiagnostics, and Clinical Examination — Three Musketeers to Differentiate Polyneuropathies

**DOI:** 10.1007/s13311-022-01211-0

**Published:** 2022-03-07

**Authors:** Natalie Winter, Alexander Grimm

**Affiliations:** 1 Neurology and Epileptology, University Hospital Tuebingen, Tuebingen, Germany; 2 Department of Neurology and Epileptology, Hertie Institute for Clinical Brain Research, University of Tuebingen, Tuebingen, Germany

**Keywords:** Nerve ultrasound, POEMS, CIDP ultrasound, Immune mediated neuropathies

Acquired demyelinating polyneuropathies consist of a variety of different disease entities including immune-mediated, hematological, inherited, and paraneoplastic-mediated neuropathies. Early diagnosis is important in order to be able to initiate the appropriate therapy. In recent years, with the help of high-resolution nerve ultrasound, progress has been made in distinguishing different mono- and polyneuropathies, in monitoring the disease course, and further understanding the underlying pathomechanisms.

In this issue of *Neurotherapeutics*, Niu et al. [1] from China present diagnostic criteria using electrophysiological and sonographic data to distinguish polyneuropathy, organomegaly, endocrinopathy, monoclonal protein, and skin changes (POEMS) syndrome from chronic inflammatory demyelinating polyneuropathy (CIDP). With this work, the authors reemphasize the value of nerve ultrasound as a diagnostic tool.

Immune-mediated neuropathies represent a heterogeneous group of peripheral nerve disorders that can be classified according to time course, predominant involvement of motor/sensory fibers, distribution of deficits, and paraclinical parameters such as nerve conduction studies, high resolution nerve ultrasound, and serum antibodies [2]. Among these, Guillain-Barré syndrome (GBS) and CIDP represent the most common subtypes with a prevalence ranging from 1–3/100,000, respectively [2].

Although electrodiagnostics and clinical examination remain the hallmark of a proper diagnosis, the rate of false positive diagnoses in immune-mediated neuropathies can be up to 47% [3] due to the similarity of differential diagnoses, which might mimic CIDP — particularly in early stages, such as transthyretin (TTR) amyloidosis or POEMS syndrome. A thorough diagnostic workup is, therefore, essential for preventing misdiagnosis and treatment delay.

Especially high-resolution nerve ultrasound adds valuable information to gain a more efficient diagnostic workup and treatment follow-up [4–6]. The most important ultrasound finding is nerve enlargement, which is seen in more than 90% of patients with immune-mediated neuropathies. The enlargement occurs mainly in nerve roots, the brachial plexus and proximal arm, and leg segments [4–6]. In addition to nerve cross-sectional area and distribution pattern, other ultrasound parameters such as nerve echogenicity play an important role.

In their work, Niu et al. [1]  present these different ultrasound features to further distinguish CIDP from POEMS. In both forms of polyneuropathy, the authors demonstrate nerve enlargement, which was significantly more pronounced in the 120 CIDP patients compared to the 34 POEMS patients. The nerve enlargement pattern, however, was different: in CIDP patients, it was distributed more proximally and focally, in line with previous publications, while POEMS patients showed more homogeneously distributed nerve alterations, analogous to our knowledge of the more homogeneous nerve conduction velocity reduction in the latter. The ratio of maximum/minimum cross-sectional area of the median nerve was significantly larger in CIDP. This was also reflected in a higher detection rate of conduction blocks and probable conduction blocks in CIDP patients (50% of CIDP patients vs none in POEMS patients). The authors propose a two-step protocol using the detection of conduction blocks and the measurement of maximum/minimum cross-sectional area of the median nerve (sensitivity 93%, specificity 79%) [1].

Apart from these findings, echo intensity was analyzed and was consistent with previous analyses by Dörner et al. [7]: POEMS patients predominantly have hypoechoic nerves, whereas in CIDP patients, both hypo- and hyperechoic nerves can be found.

The study by Niu et al. [1]  applies scientific rigor, and the large number of patients with rare diseases is one of its strengths. Ultrasound findings in particular are very consistent with the current body of knowledge in regard to the pathophysiology of CIDP and POEMS syndrome.

Analysis of nerve biopsies in 35 patients with POEMS syndrome and 26 with “typical” CIDP showed that POEMS syndrome nerve biopsies have higher rates of axonal degeneration, diffuse myelinated nerve fiber loss, and increased numbers of small epineurial blood vessels. In contrast, CIDP nerve biopsies demonstrated significantly higher rates of endoneurial inflammation, multifocal myelinated nerve fiber loss, and onion bulb formation [8].

Padua et al. [6] as well as Härtig et al. [9] were able to differentiate three types of distinct ultrasound morphologies in autoimmune neuropathies: group 1 consisted of patients with hypoechoic and enlarged nerves, group 2 individuals had hyperechoic and enlarged nerves, and the last group showed hardly any nerve enlargement with mixed echogenicity. Patients in groups 1 and 2 benefitted most from treatment with either steroids or intravenous immunoglobulins, whereas patients in group 3 did not improve under treatment with immunosuppressants. Histopathological examinations of available sural nerve biopsies obtained from patients of each category proved the theory of past inflammation and primarily axonal damage in group 3 and demonstrated signs of an ongoing immune reaction in groups 1 and 2 [9]. In addition, semiquantitative analysis of nerve echogenicity confirmed the association of higher nerve echogenicity with a more chronic and severe disease course [10].

What generally needs to be discussed in this context is the extent of the ultrasound protocol. With regard to the various polyneuropathies described in general and the different typical and atypical forms of CIDP in particular, treatment-naïve or treated polyneuropathies, a thorough and extensive ultrasound examination is needed. Especially nerve thickening and its distribution pattern must be sufficiently assessed. Too few measurement points could result in false-negative diagnoses. On the other hand, an ultrasound measurement protocol should be designed efficiently and be practical in daily clinical practice. The authors of this manuscript decided to use 10 measurement points per nerve, which might be difficult to implement in a clinician’s daily routine. Other authors have developed alternative measurement protocols for the differential diagnosis of polyneuropathies in general [11, 12] or immune-mediated polyneuropathies in particular [13]. Each of these protocols has its one strength and weaknesses, mirroring the heterogeneity of polyneuropathies. The optimization of these ultrasound protocols will remain an important and exciting task in the future. Niu et al. [1] have contributed a valuable piece of work to this endeavor.
